# Associations of Metabolites Related Salt Sensitivity of Blood Pressure and Essential Hypertension in Chinese Population: The EpiSS Study

**DOI:** 10.3390/nu17071289

**Published:** 2025-04-07

**Authors:** Xiaojun Yang, Fengxu Zhang, Bowen Zhang, Han Qi, Yunyi Xie, Wenjuan Peng, Bingxiao Li, Fuyuan Wen, Pandi Li, Yuan Sun, Aibin Qu, Ling Zhang

**Affiliations:** 1 Department of Epidemiology and Health Statistics, School of Public Health, Capital Medical University, and Beijing Key Laboratory of Environment and Aging, Beijing 100069, China; yxiaojun99@163.com (X.Y.); 13662052236@163.com (F.Z.); zhangbowen_27@163.com (B.Z.); qihan23@126.com (H.Q.); yiyi95127@126.com (Y.X.); pengwenjuan0311@126.com (W.P.); libingxiao13@163.com (B.L.); wenfy1997@ccmu.edu.cn (F.W.); lipandi1997@163.com (P.L.); sunyuankg@126.com (Y.S.); quaibin@mail.ccmu.edu.cn (A.Q.); 2 Health Management Center, Beijing Aerospace General Hospital, Beijing 100076, China

**Keywords:** salt sensitivity of blood pressure, metabolomics, essential hypertension, biomarkers, mediation analysis

## Abstract

Background: Salt sensitivity of blood pressure (SSBP) is an important risk factor for essential hypertension and cardiovascular diseases, and its metabolic mechanisms remain poorly understood. This study aimed to identify SSBP-associated metabolic biomarkers and investigate their potential mediating role in the SSBP-hypertension pathophysiology. Methods: Based on the Systematic Epidemiological Study of Salt Sensitivity (EpiSS) conducted in 2014–2016, we performed a case-control study involving 54 matched pairs of participants classified as salt-sensitive or salt-resistant with targeted metabolomics detected. Multivariable logistic regression analyses were conducted to assess the metabolites associations with SSBP and hypertension. The diagnostic performance of the model was evaluated using the receiver operating characteristic curve (ROC) analysis yielded an area under the curve (AUC) value, sensitivity, and specificity. Furthermore, the potential mediating effects of targeted metabolites on the relationship between SSBP and essential hypertension were explored. Results: Three metabolites demonstrated significant SSBP associations: L-Glutamine (OR = 0.998; 95% CI: 0.997, 0.999), PC (16:1/14:0) (OR = 1.039; 95% CI: 1.003, 1.077), and ChE (22:4) (OR = 1.115; 95% CI: 1.002, 1.240). Among them, L-Glutamine demonstrated the highest diagnostic efficiency for SSBP (AUC = 0.766; 95% CI: 0.677, 0.855). The combined model of the three metabolites slightly improved diagnostic efficiency (AUC = 0.788; 95% CI: 0.703, 0.874). L-Glutamine and Cer (d18:0/24:1) were identified as potential protective factors against essential hypertension (*p* < 0.05). Mediation analyses further indicated that L-Glutamine partially mediated the relationship between SSBP and essential hypertension, demonstrating a suppressive effect. Conclusions: This study identified L-Glutamine as both a diagnostic biomarker for SSBP and a metabolic modulator attenuating hypertension risk, providing insights for early SSBP screening and the pathways governing SSBP progression to overt hypertension.

## 1. Introduction

Essential hypertension is the main risk factor for cardiovascular and kidney pathologies, and it represents a major global public health challenge associated with premature mortality [1]. Substantial epidemiological evidence has established causal relationships between dietary sodium consumption and blood pressure (BP) regulation [2,3]. The American Heart Association formally defines salt sensitivity of blood pressure (SSBP) as the physiological characteristic of interindividual variability in BP response to dietary sodium fluctuation [4]. Based on changes in BP following salt load and diuresis, the individuals can be divided into salt-sensitive (SS)and salt-resistant (SR) [5]. Under high sodium conditions, SS individuals demonstrate compromised cardiovascular homeostasis, where elevated BP serves as a maladaptive mechanism to enhance sodium excretion, consequently predisposing to hypertension development [4]. The Genetic Epidemiology Network of Salt Sensitivity (GenSalt) study found that those individuals with high salt sensitivity have a 43% higher risk of developing hypertension compared to those with low salt sensitivity [6]. This risk differential underscores the clinical imperative for early SS identification to enable precision prevention strategies and targeted hypertension management. Determining SSBP not only enables early intervention but also facilitates personalized treatment strategies, such as customized dietary guidelines and targeted anti-hypertensive therapies, which can effectively reduce the risk of cardiovascular diseases associated with salt sensitivity. Current diagnostic approaches rely on dietary salt load tests or acute intravenous saline loading [7,8,9], methodologies constrained by substantial resource requirements and limited scalability in clinical practice. The dietary salt load tests require strict adherence to high-salt or low-salt interventions over several weeks, necessitating multiple ambulatory blood pressure monitoring sessions that compromise patient compliance and prolong diagnostic confirmation. Similarly, the acute intravenous salt load test requires saline infusion and diuretic-induced sodium depletion, carries inherent risks of volume overload and electrolyte disturbances, particularly in vulnerable populations. Moreover, these methodologies demand specialized clinical monitoring and complex technology, which further limits its feasibility for large-scale application. This diagnostic impasse highlights the critical need for novel SS biomarkers that enable rapid, accurate risk stratification in routine clinical settings.

The emergence of high-throughput metabolomics has revolutionized biomarker discovery, enabling systematic interrogation of metabolic perturbations underlying disease pathophysiology [10,11]. As terminal effectors in the central dogma of biology, metabolites integrate genomic, transcriptomic, and proteomic information, serving as sensitive indicators of phenotypic manifestations [12]. This positional advantage makes metabolomics particularly suited for detecting subclinical SSBP signatures through dynamic metabolic flux analysis prior to overt hypertension onset. Existing SSBP metabolomic investigations have predominantly utilized rodent models [13] and urine metabolite profiling [14]. Although these approaches have identified candidate biomarkers, their translational utility remains constrained by biological matrix limitations. Urine metabolites primarily reflect renal handling, whereas plasma metabolites provide systemic pathophysiological insights. Consequently, plasma-based metabolomic profiling emerges as a critical unmet need, offering whole-body metabolic readouts through circulating biomarkers that integrate multi-organ system interactions. Essential hypertension is recognized as a metabolic disorder, and numerous studies have demonstrated a strong association between metabolites and the risk of hypertension [15,16]. Current mechanistic investigations into SSBP-hypertension metabolic crosstalk remain disproportionately focused on urinary biomarkers. Previous studies have demonstrated that variations in homocysteine levels are significantly correlated with changes in diastolic blood pressure [17]. Furthermore, SSBP-associated metabolites (serine, 2-methylbutyrylcarnitine, isoleucine) demonstrate hypertension-predictive capacity independent of traditional risk factors [14]. Conducting metabolomics research on blood samples can further elucidate the potential metabolic mechanisms underlying the relationship between SSBP and hypertension. Our prior untargeted metabolomics study identified 39 differentially abundant metabolites distinguishing SS from SR phenotypes [18], with sphingolipid species constituting 40% of the signature. Pathway enrichment analysis revealed predominant involvement of sphingolipid signaling, pyruvate metabolism, and arginine biosynthesis. While untargeted metabolomics approaches can discover metabolic perturbations, their technical limitations in compound identification rates and quantification accuracy hinder clinical translation. Targeted metabolomics overcomes these limitations through predefined multiple reaction monitoring assays, offering superior sensitivity and reliability. Targeted metabolomics can further validate the accuracy of biomarkers identified through untargeted metabolomics for SSBP diagnosis and facilitate the exploration of how metabolites mediate the relationship between SSBP and hypertension.

This study employs targeted metabolomics in an independent validation population to confirm prior untargeted findings, construct a SSBP diagnostic model and elucidate metabolic mediation effects. Through causal mediation frameworks, we further quantify metabolite-specific mediation effects (proportion mediated) in the SSBP-hypertension continuum, advancing both mechanistic understanding and precision prevention strategies.

## 2. Materials and Methods

### 2.1. Participants

All participants were recruited from the Systematic Epidemiological Cohort Study of Salt Sensitivity Blood Pressure (EpiSS), a cohort established between 2014–2016, which included 2057 individuals. The study aimed to investigate environmental and genetic risk factors associated with SSBP and their implications for major chronic diseases. Detailed information about the cohort has been previously published [7,19], and it is registered in the Chinese Clinical Trial Registry (Registration No: ChiCTR-EOC-16009980). Participants were aged 35–70 years. Data on participants’ demographic characteristics, lifestyle factors, medical history, and medication use were systematically collected. The exclusion criteria included pregnancy, kidney disease, malignant tumors, stage 2 or higher hypertension, secondary hypertension, and adherence to a low-sodium diet, as the latter significantly influences BP compared to a typical diet [20].

In the present study, a case-control study was employed. A total of 54 matched pairs of participants (SS vs. SR) were recruited and matched 1:1 by age (±5 years) and gender. The study protocol was approved by the Ethics Committee of Capital Medical University (Approval No: Z2023SY025), and all participants provided written informed consent.

### 2.2. Determination of Salt Sensitivity of Blood Pressure

The determination of SSBP in the EpiSS cohort was based on modified Sullivan’s Acute Oral Saline Load and Diuresis Shrinkage Test (MSAOSL-DST) [7]. All participants were required to fast for at least 8 h prior to the MSAOL-DST test, and no medications were allowed on the day of the test to avoid any potential impact on SSBP assessment. As described in a previous study [7], the MSAOSL-DST was performed according to the following steps: (i) Baseline blood pressure (BP_0_) was measured after 15 min of quiet sitting. (ii) Participants were asked to drink 1 L of 0.9% saline solution within 30 min, and blood pressure (BP_1_) was measured two hours later, which was called the acute saline load period. (iii) Blood pressure (BP_2_) was measured two hours after oral administration of 40 mg of furosemide, and this period was called the diuresis shrinkage period. At each stage, BP was measured three times, and the average value was calculated. The mean arterial pressure (MAP) was calculated using the formula MAP = (SBP + 2 × DBP)/3 [21]. The ΔMAP_1_ was calculated as the MAP_1_ minus the MAP_0_. The ΔMAP_2_ was calculated as the MAP_2_ minus the MAP_1_. Individuals with ΔMAP_1_ ≥ 5 mmHg or ΔMAP_2_ ≤ −10 mmHg were defined as SS. In other cases, they were considered salt-insensitive (or SR).

### 2.3. Data Collection

Standardized questionnaires were employed to collect general characteristics, behavior, and medical history. BP was measured in the morning in triplicate by a trained observer using an OMRON digital BP monitor (Omron Healthcare, Kyoto, Japan). Participants were seated in a relaxed position after a 15-min rest period and had refrained from caffeine, food, physical activity, smoking, and alcohol for 12 h prior to the clinic visit. According to the 2018 Chinese guidelines for the management of hypertension [22], essential hypertension is defined as systolic blood pressure (SBP) ≥ 140 mmHg and/or diastolic blood pressure (DBP) ≥ 90 mmHg without the use of anti-hypertensive drugs. Subjects with a BP < 140/90 mmHg but having hypertensive history and currently are taking anti-hypertensive medication should also be diagnosed as hypertensives.

Peripheral venous blood of participants was collected in the morning after an overnight fast. Serum and plasma samples were obtained through centrifugation and stored at −80 °C. Plasma samples were subsequently used for targeted metabolomics analysis. Serum samples were used for biochemical examination, including the assessment of total cholesterol (TC), triglyceride (TG), high-density lipoprotein cholesterol (HDL-C), low-density lipoprotein cholesterol (LDL-C), and fasting blood glucose (FBG) levels. The 24-h urine samples were collected from participants within one week after SSBP determination, during which participants maintained their normal diet. Urine samples with a volume greater than 500 mL were considered valid. Twenty-four-hour urinary sodium excretion (24hUNa) and 24-h urine potassium excretion (24hUK) were measured using ion-selective electrode techniques and analyzed with a Cobas C8000 (Roche, Basel, Switzerland).

### 2.4. Metabolomic Profiling and Quality Control

Considering the practical application value and clinical translational potential of metabolites, we prioritized those with statistical significance and strong supporting evidence from the literature for targeted verification. Based on the results of published untargeted metabolomics studies [18], we selected 13 of 39 SSBP differentially expressed metabolites for targeted metabolomics research by referring to relevant metabolite reports, principal component analysis (PCA), the least absolute shrinkage and selection operator (LASSO) regression model, and the random forest method. Accurate quantification of metabolites in plasma samples stored at −80 °C was performed using high-performance liquid chromatography-tandem mass spectrometry (HPLC-MS/MS). The isotope internal standard single point method was used to quantify lipid metabolites, including Phosphatidylcholine (16:1/14:0) [PC (16:1/14:0)], Cholesteryl ester (22:5n6) [ChE (22:5n6)], Acylcarnitine (20:3) [AcCa (20:3)], Cholesteryl ester (22:5n3) [ChE (22:5n3)], Cholesteryl ester (22:4) [ChE (22:4)], Ceramide (d18:0/24:1) [Cer (d18:0/24:1)], and Triacylglycerol (54:6) [TAG (54:6)]. Polar metabolites, including N(6)-methyllysine, L-Glutamine, L-Lactic acid, and L-Malic acid, were quantified using the isotope internal standard method. Seven metabolites were employed as internal standards for the quantification of the 11 metabolites described above. Detailed information about the internal standards is shown in Supplementary Table S1. Additionally, 13(S)-Hydroxy-octadecadienoic acid [13(S)-HODE] and 9(S)-Hydroxy-octadecadienoic acid [9(S)-HODE)] were quantified using the external standard method. Due to the unique properties of lipid molecules, there are no corresponding limit of detection (LOD) and limit of quantification (LOQ) available. Detailed information regarding the LOD and LOQ for the remaining six metabolites analyzed by HPLC-MS/MS is provided in Supplementary Table S2. Quality control of the metabolite data was performed by assessing the percentage coefficient of variation (CV%) of the quantitative concentration values of the target substances in the quality control samples. A CV% value below 20% is generally considered acceptable, indicating that the metabolites have stable properties and can be accurately and controllably quantified.

Data acquisition was conducted using the selective reaction monitoring mode (SRM) [23]. This method reduced background signals from complex components by selecting specific parent-product ion pairs, thereby enhancing the detection sensitivity for low-abundance metabolites. Furthermore, it minimized daily instrument calibration variability and batch effects on quantitative results, ensuring the reliability and reproducibility of the findings. Metabolite peaks were quantified by measuring the area under the curve, with the original values representing the raw area counts. The detection values for all 13 metabolites were above the detection limits, and the CV% for the quality control samples was below 12%, indicating that the metabolite detection results exhibit good accuracy and reliability.

### 2.5. Statistical Methods

The distribution of continuous variables was characterized in terms of the mean and standard deviation (SD), while categorical variables were expressed as numerical values and percentages (%). A two-sided *p* < 0.05 was considered statistically significant. Paired *t*-test was used to analyze the differences between groups for normally distributed data, while the Wilcoxon signed-rank test was used for non-normally distributed or ranked variables. McNemar’s test was used to compare the categorical variables. Skewed metabolite data were log-transformed and analyzed statistically. Sample size estimation and power analysis were conducted using MetaboAnalyst 6.0, indicating that 22 participants per group would achieve 90% power at α = 0.05. Our study met the required sample size for metabolomics analysis. The expression levels of metabolites in the SS and SR group were visualized by violin plots. The associations between metabolites and SSBP, ΔMAP_1_, and ΔMAP_2_ were assessed using multivariate logistic regression models or multivariate linear regression models, adjusting for age, sex, body mass index (BMI), smoking, LDL-C, baseline SBP, and family history of hypertension. In the sensitivity analysis, we constructed four models based on the primary model to assess the robustness of our findings. Model 1 adjusted for the same variables as the primary model, excluding baseline SBP. Model 2 replaced baseline SBP with baseline DBP in the adjustment. Model 3 included baseline DBP as an additional covariate alongside the variables in the primary model. Model 4 substituted baseline SBP with baseline MAP in the adjustment. Odds ratios (ORs) or beta coefficients (*β*) and 95% confidence intervals (95% CIs) were used to assess the relationships between metabolites and SSBP, ΔMAP_1_, and ΔMAP_2_. Receiver operating characteristic (ROC) curve analysis was performed, and the area under the curve (AUC), sensitivity, and specificity were calculated to further identify metabolic biomarkers of SSBP. Additionally, multivariate logistic regression analysis was performed to explore the association between metabolites and essential hypertension, with essential hypertension as the dependent variable, adjusting for covariables including age, sex, BMI, smoking, LDL-C, HDL-C, and family history of hypertension. Statistical analysis was performed using SPSS 25.0 (SPSS, Inc., Chicago, IL, USA) and R software (version 4.3.3).

A two-step mediation analysis was conducted using the R package “Mediation” to identify and elucidate the potential mediating effect of SSBP-related metabolites between SSBP and essential hypertension. All mediation analyses were adjusted for potential confounding variables, including age, sex, and family history of hypertension. The mediation model was constructed based on the guidance of Kenny et al. [24,25], as shown in Supplementary Figure S1. In the model, X, M, and Y represent the independent variable (SSBP), mediating factor (metabolites), and dependent variable (essential hypertension), respectively. The coefficient c represents the total effect of X on Y, c’ represents the direct effect of X on Y after adjusting M, and a*b represents the indirect effect of M. In the mediation model, when the direct and indirect effects of the independent variable on the dependent variable have the same direction, it is referred to as a consistent mediation model, with the ratio of the indirect effect to the total effect is a*b/c. Additionally, a suppression effect occurs when the direct and indirect effects have opposite signs, characterizing an inconsistent mediation model. In such cases, a*b represents an estimate of the suppression effect. We performed 1000 iterations, and *p* < 0.05 was considered statistically significant.

## 3. Results

### 3.1. Basic Characteristics of the Participants

The characteristics of the participants in the targeted metabolomics study are summarized in Table 1. A total of 108 participants were enrolled, with a mean age (±SD) of 58.85 ± 6.89 years, and 60 (55.6%) were female. Except for LDL-C (*p* = 0.023), SBP (*p* = 0.014), DBP (*p* = 0.002), MAP (*p* = 0.001), ΔMAP_1_ (*p* < 0.001), and ΔMAP_2_ (*p* = 0.005), the basic characteristics of the SS and SR groups were balanced. The expression levels of 13 metabolites in the SS and SR groups are presented in Supplementary Figure S2. The univariate analysis revealed no significant differences in the expression levels of other plasma metabolites between the two groups, except for L-Glutamine and TAG (54:6) (*p* < 0.05). The plasma concentrations of L-Glutamine and TAG (54:6) in the SS group were significantly lower compared to those in the SR group.

### 3.2. Association Between Metabolites and SSBP

As shown in Table 2, multivariate logistic regression analysis was conducted to assess the associations between metabolites and SSBP, adjusting for age, sex, BMI, smoking, LDL-C, baseline SBP, and family history of hypertension. We found that L-Glutamine was a potential protective factor for SSBP (OR = 0.998; 95% CI: 0.997, 0.999), consistent with previous untargeted metabolomics studies [18], while PC (16:1/14:0) and ChE (22:4) were identified as risk factors for SSBP (OR = 1.039; 95% CI: 1.003, 1.077 and OR = 1.115; 95% CI: 1.002, 1.240). After FDR adjustment, the association between L-Glutamine and SSBP remained significant (Supplementary Table S3). L-Glutamine possesses antioxidant properties that mitigate oxidative stress, potentially offering cardiovascular protection and reducing the risk of SSBP. PC (16:1/14:0) and ChE (22:4) may contribute to an elevated risk of cardiovascular diseases by facilitating lipid accumulation and foam cell formation. In addition, we also analyzed the relationships between these metabolites and mean arterial pressure change during the acute saline load period and diuresis shrinkage period. However, after adjusting for age, sex, BMI, smoking, LDL-C, baseline SBP, and family history of hypertension, multivariate linear regression analysis showed no statistically significant associations between metabolites and ΔMAP_1_ (*p* ≥ 0.05) or ΔMAP_2_ (*p* ≥ 0.05). Furthermore, in the sensitivity analysis, when adjusting for either baseline DBP alone or both baseline SBP and DBP, only L-Glutamine remained statistically significant in its association with SSBP. However, the statistical effect sizes for PC (16:1/14:0) and ChE (22:4) were consistent with those observed in the main model (Supplementary Table S4). This trend might be explained by the relatively limited sample size, which may result in borderline statistical significance. Nevertheless, these findings further provide additional support for the robustness of our results.

### 3.3. Evaluation of the Value of Metabolites in Diagnosing SSBP

**Figure 1:**
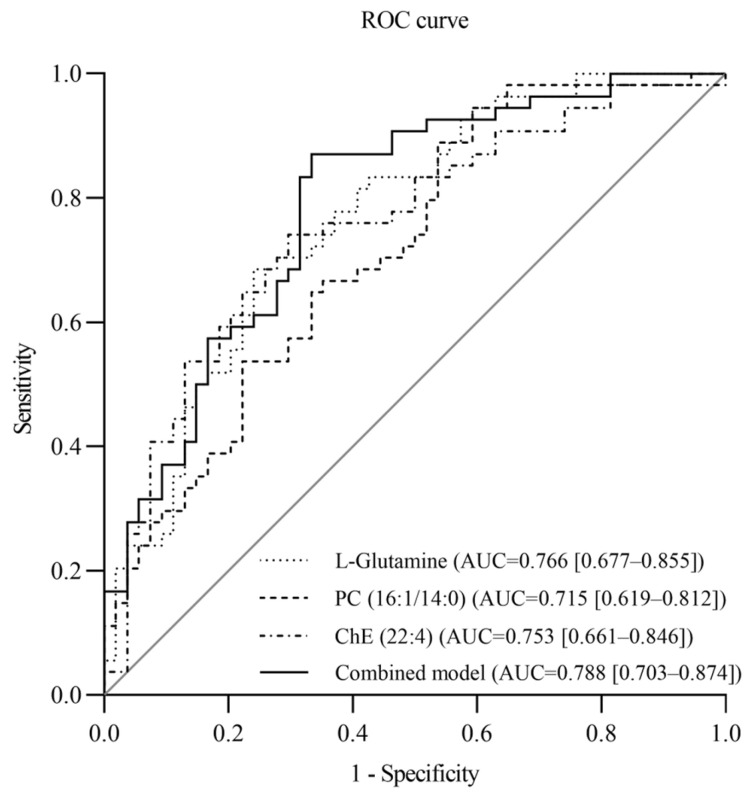
ROC curves of the single-metabolite model and combined model for the diagnosis of SSBP risk. ROC, receiver operating characteristic; AUC, the area under the ROC curve.

Based on the observed associations between specific metabolites and SSBP, we selected the key metabolites for ROC analysis to evaluate their diagnostic potential for SSBP. ROC analysis was performed to assess the diagnostic efficiency of a single-metabolite model for SSBP, adjusting for age, sex, BMI, smoking, LDL-C, baseline SBP, and family history of hypertension, and it was found that L-Glutamine exhibited the highest diagnostic efficiency for SSBP (Supplementary Table S5). The area under the curve of the L-Glutamine level was 0.766 (95% CI: 0.677, 0.855), with a sensitivity and specificity of 75.9% and 68.5%, respectively. The AUCs of PC (16:1/14:0) and ChE (22:4) were 0.715 (95% CI: 0.619, 0.812) and 0.753 (95% CI: 0.661, 0.846), respectively. Furthermore, compared with those of the single-metabolite model, the diagnostic efficiencies of the combined model constructed with L-Glutamine, PC (16:1/14:0), and ChE (22:4) were slightly greater, with an AUC, sensitivity, and specificity of 0.788 (95% CI: 0.703, 0.874), 66.7%, and 87.0%, respectively (Figure 1).

### 3.4. The Effect of Metabolites on Essential Hypertension

**Figure 2:**
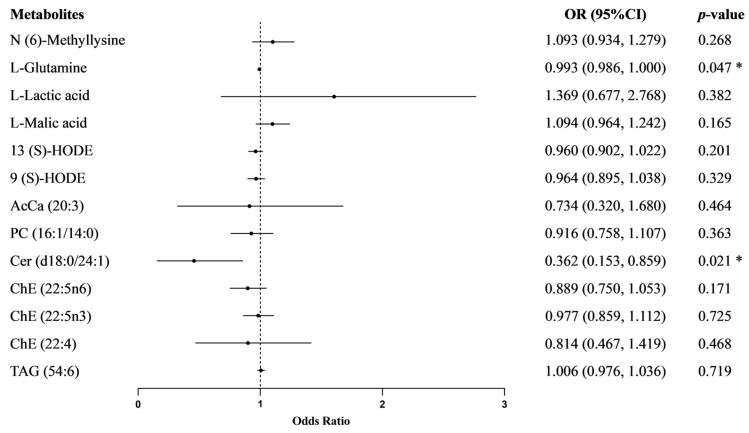
Multivariate logistic regression analysis of the associations between metabolites and essential hypertension. *, *p* < 0.05.

We conducted multivariate logistic regression analysis to assess the associations between metabolites and essential hypertension, as displayed in Figure 2. After adjusting for age, sex, BMI, smoking, LDL-C, HDL-C, and family history of hypertension, we found that L-Glutamine (OR = 0.993; 95% CI: 0.986, 1.000) and Cer (d18:0/24:1) (OR = 0.362; 95% CI: 0.153, 0.859) may serve as potential protective factors for essential hypertension. The associations between the other 11 metabolites and essential hypertension were not statistically significant (*p* ≥ 0.05). L-Glutamine may exert a protective effect on BP regulation and hypertension risk reduction by enhancing endothelial cell function and exhibiting antioxidant and anti-inflammatory properties. Ceramide (Cer) may be a key regulator of BP, as elevated levels have been strongly associated with cerebrovascular diseases, including stroke and cerebral small vessel disease, indicating its potential impact on hypertension.

### 3.5. Mediating Effect

**Figure 3:**
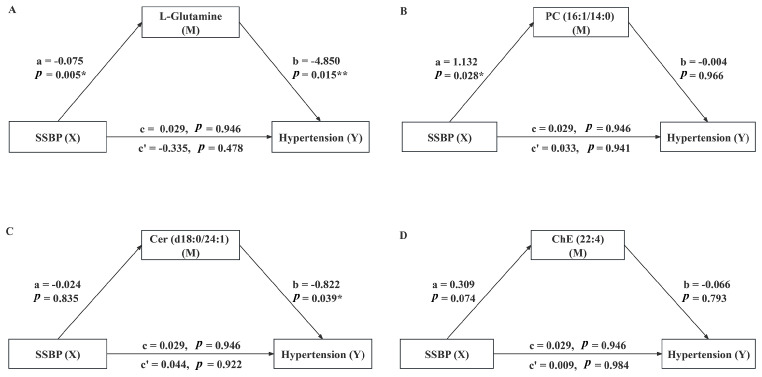
The mediating effect of metabolites on the relationship between SSBP and hypertension. (**A**) L-Glutamine mediation model; (**B**) PC (16:1/14:0) mediation model; (**C**) Cer (d18:0/24:1) mediation model; (**D**) ChE (22:4) mediation model; a, effect of SSBP on hypertension; b, effect of metabolites on hypertension; c, total effect of SSBP on hypertension; c’, direct effect of SSBP on hypertension. *, *p* < 0.05; **, *p* < 0.01.

The metabolites associated with SSBP or essential hypertension were selected for mediation analysis to investigate whether these metabolites mediate the relationship between SSBP and the development of hypertension. After adjusting for age, sex, BMI, LDL-C, and family history of hypertension, the mediation model exploring the role of metabolites in the relationship between SSBP and essential hypertension is depicted in Figure 3. SSBP was negatively correlated with L-Glutamine (a = −0.075, *p* = 0.005), and L-Glutamine was significantly negatively correlated with essential hypertension (b = −4.850, *p* = 0.015). In comparison, the direct effect of SSBP on essential hypertension was not significant (c’ = −0.335, *p* ≥ 0.05). The indirect effect coefficient for L-Glutamine was 0.364 (a*b), which opposed the direct effect coefficient, suggesting that the mediating effect of L-Glutamine in the relationship between SSBP and essential hypertension was a suppressive effect [25]. The mediating effect of other metabolites between SSBP and essential hypertension was not statistically significant (*p* ≥ 0.05).

## 4. Discussion

Previous studies have identified L-Glutamine as a biomarker for SSBP based on untargeted metabolomics analysis [18]. In our study, further targeted validation confirmed a negative correlation between L-Glutamine and SSBP, suggesting its potential role as a protective factor against this condition. Similar findings were observed in the sensitivity analysis after excluding baseline SBP and including DBP. L-Glutamine, the most abundant and versatile amino acid in the human body, plays critical roles in nitrogen exchange, intermediary metabolism, immune response modulation, and the maintenance of pH balance across various organs [26,27,28]. L-Glutamine plays a fundamental role in cardiovascular physiology and pathology [29]. Mansour et al. reported that oral L-Glutamine supplementation reduced SBP among individuals with type 2 diabetes [30]. The reduction in BP caused by L-Glutamine may be due to increased L-arginine synthesis, which in turn leads to increased NO production [31]. Increased NO levels promote vascular relaxation and the recruitment of tissue-supplying arteries [32]. Consequently, it is reasonable to conclude that L-Glutamine serves as a protective factor against SS and essential hypertension. Additionally, this study also identified PC (16:1/14:0) and ChE (22:4) as potential risk factors for SSBP. Previous studies have indicated that elevated levels of cholesterol esters and glycerophosphocholine (GPC) are associated with an increased risk of cardiovascular disease [33,34]. Excessive cholesterol esterification or impaired cholesterol release contribute to the accumulation of cholesterol esters (CEs) within cytoplasmic lipid droplets, leading to the formation of foam cells [35]. The formation of macrophage foam cells within the arterial intima is a characteristic feature of the early stages of atherosclerotic lesions. The Framingham Heart Study found that cholesterol ester 16:1 mediated the relationship between the ideal cardiovascular health (CVH) score with incident atrial fibrillation (AF) [36]. Furthermore, previous studies have demonstrated that decreased choline intake can reduce cardiovascular risk by lowering plasma homocysteine levels [37]. On the other hand, chronic choline supplementation in mice was found to elevate trimethylamine N-oxide (TMAO) levels and was linked to atherosclerosis development in a murine model [38]. Our analysis supports these findings. For instance, SS individuals had higher levels of cholesterol ester (22:5n6) and glycerophosphocholine (16:1/14:0).

Currently, existing diagnostic methods for SSBP are costly and labor-intensive, highlighting the need for easily measurable and rapid biomarkers for SSBP to facilitate large-scale clinical diagnosis. Although some studies have constructed SSBP diagnostic models based on genomics and transcriptomics [19,21,39], research in the field of metabolomics remains limited. Peng et al. reported that the combination of lnc-IGSF3-1:1 + SCOC-AS1 + SLC8A1-AS1 demonstrated optimal diagnostic performance for SSBP in both hypertensive (AUC = 0.853) and normotensive groups (AUC = 0.873) [19]. Qi et al. found that the diagnostic model of SSBP constructed by hsa-miR-361-5p had higher efficiency, with an AUC of 0.793 (95%CI: 0.698, 0.888) [39]. In this study, we constructed a diagnostic model of SSBP based on SSBP-related metabolites. Among the single-metabolite models, L-Glutamine (AUC = 0.766; 95% CI: 0.677, 0.855) emerged as the most effective diagnostic marker for SSBP. L-Glutamine has also demonstrated higher diagnostic performance in previous untargeted metabolomics studies, with an AUC of 0.88 (95% CI: 0.78, 0.97) [18]. Furthermore, L-Glutamine, an amino acid, can be detected using widely available and cost-effective mass spectrometry techniques. The combined model based on L-Glutamine, PC (16:1/14:0), and ChE (22:4) showed a slight improvement in the diagnostic ability of SSBP, with an AUC of 0.788 (95% CI: 0.703, 0.874). Based on previous studies demonstrating the strong diagnostic performance of L-Glutamine and considering its cost-effectiveness, we recommend the use of a single-metabolite model for SSBP diagnosis. However, further validation studies in larger, more diverse populations are necessary before clinical implementation.

We further hypothesized that SSBP was associated with essential hypertension by influencing metabolite expression levels. Our findings suggest that L-Glutamine and Cer (d18:0/24:1) may serve as protective factors against essential hypertension. However, the inverse association between L-Glutamine and essential hypertension exhibits only borderline statistical significance. Although the *p*-value is slightly below the conventional threshold of 0.05, the OR is close to 1, suggesting a weak effect, which may be attributed to the limited sample size. Notably, existing studies have reported inconsistent findings regarding the role of L-Glutamine in cardiovascular diseases. The vascular endothelium serves as a critical regulator of blood vessel structure and function. Previous studies have highlighted a central role for glutamine in promoting endothelial cell function, influencing BP regulation by modulating vascular permeability, arterial tone, and the proliferation and migration of smooth muscle cells [29]. Yang et al. reported that compared with hypertensive rats fed a high-salt diet (SBP: 165.6 mmHg, DBP: 125.1 mmHg), hypertensive rats receiving high-dose L-Glutamine supplementation exhibited a significant prevention of SBP increase (152.6 mmHg) and a slight mitigation of DBP increase (120.9 mmHg) [40]. In contrast, Egnatchik et al. found elevated systemic and pulmonary-specific glutamine metabolism levels in the pulmonary vasculature of the pulmonary arterial hypertension (PAH) group [41]. Similarly, Bertero et al. observed an upregulation of glutaminase expression in lung tissues from patients with PAH [42]. Although L-Glutamine appears to be a potential protective factor against essential hypertension in this study, this finding requires further validation in larger, multicenter populations to confirm its robustness and clinical relevance. Furthermore, sphingolipid metabolic pathways may play a crucial role in regulating blood pressure [43]. The intermediate of the sphingolipid pathway is ceramide [44]. Ceramide, an essential component of sphingolipids, serves as an intracellular signal of free fatty acid abundance and plays a crucial role in the pathophysiology of hypertension by modulating lipid burden responses [45]. Increasing evidence highlights a robust association between elevated ceramide levels and cerebrovascular diseases, particularly stroke and cerebral small vessel disease (CSVD) [46]. Mantovani et al. found that ceramides, particularly Cer (d18:1/24:1), were independently associated with the presence of inducible myocardial ischemia, emphasizing the potential role of ceramides in the pathophysiology of adverse cardiovascular events [47]. Currently, ceramides have been confirmed as accurate biomarkers for adverse cardiovascular disease outcomes [48]. Lee et al. found that sphingosine-1-phosphate and very-long-chain ceramide levels were significantly reduced in patients with acute ischemic stroke, whereas long-chain ceramide levels were significantly elevated [49]. This suggests that ceramides of different chain lengths may play distinct roles in the pathophysiological processes of acute ischemic stroke. In contrast, Lin et al. identified four metabolites (indole-3-acrylic acid, 3-hydroxybenzoic acid, PC 18:0p/20:4, and PG 18:1/18:2) that were significantly associated with BP through untargeted metabolomics and lipidomics analysis [50]. These metabolites may further support the role of metabolic alterations in BP regulation and provide complementary insights into the molecular pathways involved in hypertension. Further mediation analysis revealed that L-Glutamine mediated the relationship between SSBP and hypertension, exhibiting a masking effect that resulted in opposing directions for the direct and indirect effects. However, Kennedy et al. demonstrated that the mediation and suppression models are statistically equivalent [25]. This mediating effect can be interpreted as L-Glutamine acting as a suppressor variable in the pathway between SSBP and essential hypertension. Its presence enhances the strength of the direct effect, leading to an opposing relationship between the direct and indirect effects, thereby masking or suppressing the total effect.

Our research has several advantages. First, this is the first study to explore metabolic biomarkers of SSBP based on targeted metabolomics, offering novel evidence to establish accurate and reliable SSBP assessment standards, facilitate early identification of salt-sensitive individuals, and enable personalized treatment strategies. Second, we further investigated the effect of SSBP-related metabolites between SSBP and essential hypertension, providing new insights into the metabolic pathways through which SSBP influences hypertension pathophysiology. Precision treatment strategies targeting these metabolites could assist clinicians in managing BP effectively and reducing adverse outcomes related to SSBP. Several limitations should be noted. Firstly, the high cost of metabolomic detection limited both the sample size and the number of metabolites analyzed in this study. Potential selection bias in case-control matching, along with the limitations of the cross-sectional study design, restricts the ability to draw causal conclusions. Nevertheless, these findings lay a solid foundation for identifying SSBP biomarkers. Future large-scale, multicenter longitudinal studies with more stringent control of confounding factors are required to validate these metabolites and establish causal relationships between metabolites and disease. Secondly, various confounding factors in this study, including dietary habits, physical activity, lifestyle, may influence metabolite levels and potentially affect the observed associations. Although we adjusted for major covariates, residual confounding cannot be completely excluded. Future studies should incorporate detailed dietary assessments, physical activity evaluations, and validation in independent cohorts, to further strengthen the robustness and generalizability of our findings. Thirdly, kidney function may significantly influence the levels of metabolites such as plasma L-Glutamine and act as a confounding factor in the analysis of the association between metabolites and SSBP. Due to limitations in the available data, this study did not measure kidney function indicators such as serum creatinine or estimated glomerular filtration rate (eGFR). Future research should incorporate kidney function data to better account for its potential impact and provide more accurate interpretations of the observed associations. Finally, although our findings indicate that L-Glutamine mediates the relationship between SSBP and essential hypertension, the underlying pathogenic mechanisms require further validation through animal studies.

## 5. Conclusions

In summary, this targeted metabolomics study identified L-Glutamine, PC (16:1/14:0), and ChE (22:4) as potential biomarkers for SSBP. While the combined model demonstrated slightly higher diagnostic performance compared to the single-metabolite model, the latter is recommended due to its cost-effectiveness and practical diagnostic utility. However, further validation studies in larger and more diverse populations are necessary before the clinical implementation of the SSBP diagnostic model. Moreover, L-Glutamine and Cer (d18:0/24:1) were found to be associated with essential hypertension, with L-Glutamine showing a borderline statistical significance in its relationship with hypertension. Mediation analysis further revealed that L-Glutamine may mediate the relationship between SSBP and hypertension, highlighting its potential role in the pathophysiology of hypertension. However, these findings should be interpreted with caution. Larger-scale studies are required to further investigate the specific biological mechanisms linking these metabolites to SSBP and hypertension, with the goal of providing stronger evidence for early prevention and precision treatment.

## Figures and Tables

**Table 1:** Comparison of baseline characteristics between the salt-sensitive and salt-resistant groups.

Variables	Total (*n* = 108)	SS (*n* = 54)	SR (*n* = 54)	*p*
Age (years)	58.85 ± 6.89	58.69 ± 7.44	59.02 ± 6.36	0.805 ^a^
Female (*n*, %)	60 (55.56)	31 (57.41)	29 (53.70)	0.851 ^c^
BMI (kg/m^2^)	25.88 ± 3.53	25.64 ± 3.71	26.12 ± 3.36	0.503 ^a^
WHR	0.90 ± 0.06	0.90 ± 0.61	0.90 ± 0.06	0.541 ^a^
SBP (mmHg)	122.33 ± 18.39	118.17 ± 18.05	128.49 ± 17.94	0.014 ^a^
DBP (mmHg)	77.29 ± 10.11	74.49 ± 9.57	80.09 ± 9.95	0.002 ^a^
MAP (mmHg)	92.30 ± 11.60	89.05 ± 11.30	95.56 ± 11.07	0.001 ^a^
ΔMAP_1_ (mmHg)	3.15 ± 6.03	7.23 ± 4.15	−0.94 ± 4.71	<0.001 ^a^
ΔMAP_2_ (mmHg)	−1.28 ± 6.76	−3.27 ± 7.14	0.72 ± 5.76	0.005 ^a^
FBG (mmol/L)	6.09 ± 2.09	6.41 ± 2.49	5.77 ± 1.55	0.203 ^b^
TC (mmol/L)	5.13 ± 1.04	4.94 ± 1.09	5.31 ± 0.96	0.052 ^a^
TG (mmol/L)	2.04 ± 1.34	2.08 ± 1.52	2.01 ± 1.13	0.914 ^b^
LDL-C (mmol/L)	2.62 ± 0.69	2.48 ± 0.65	2.76 ± 0.71	0.023 ^a^
HDL-C (mmol/L)	1.30 ± 0.52	1.42 ± 0.69	1.18 ± 0.23	0.160 ^b^
24hUNa (g/day)	3.24 ± 1.68	3.47 ± 1.61	3.01 ± 1.74	0.198 ^b^
24hUK (g/day)	1.86 ± 1.06	2.06 ± 1.21	1.66 ± 0.84	0.051 ^b^
Smoking (yes, %)	26 (24.07)	16 (29.63)	10 (18.52)	0.286 ^c^
Drinking (yes, %)	37 (34.26)	23 (42.59)	14 (25.93)	0.078 ^c^
Family history of hypertension (*n*, %)	50 (46.30)	24 (44.44)	26 (48.15)	0.845 ^c^

Abbreviations: SS, salt sensitivity; SR, salt resistance; BMI, body mass index; WHR, waist-to-hip ratio; SBP, systolic blood pressure; DBP, diastolic blood pressure; MAP, mean arterial pressure; ΔMAP_1_, mean arterial pressure change during the acute saline load period; ΔMAP_2_, mean arterial pressure change during the diuresis shrinkage period; FBG, fasting blood glucose; TC, cholesterol; TG, triglycerides; LDL-C, low-density lipoprotein cholesterol; HDL-C, high-density lipoprotein cholesterol; 24hUNa, 24-h urinary sodium excretion; 24hUK, 24-h urinary potassium excretion; ^a^, Paired *t*-test; ^b^, Wilcoxon signed-rank test; ^c^, McNemar’s test.

**Table 2:** Associations of metabolites with SSBP, ∆MAP_1_ during the saline load period and ∆MAP_2_ during the diuresis shrinkage period.

Metabolites	SSBP	ΔMAP_1_ (mmHg)	ΔMAP_2_ (mmHg)
	OR (95% CI)	*β* (95% CI)	*β* (95% CI)
N(6)-Methyllysine	1.020 (0.987, 1.053)	0.259 (−0.089, 0.608)	−0.121 (−0.561, 0.319)
L-Glutamine	0.998 (0.997, 0.999) **	−0.010 (−0.024, 0.004)	0.011 (−0.007, 0.028)
L-Lactic acid	1.035 (0.897, 1.195)	0.728 (−0.830, 2.287)	0.001 (−1.958, 1.960)
L-Malic acid	1.018 (0.992, 1.044)	−0.001 (−0.282, 0.280)	−0.213 (−0.562, 0.135)
13(S)-HODE	0.996 (0.983, 1.008)	0.043 (−0.095, 0.180)	0.163 (−0.006, 0.333)
9(S)-HODE	0.999 (0.984, 1.014)	0.066 (−0.100, 0.233)	0.146 (−0.061, 0.352)
AcCa (20:3)	0.929 (0.778, 1.109)	−0.736 (−2.661, 1.189)	0.856 (−1.553, 3.266)
PC (16:1/14:0)	1.039 (1.003, 1.077) *	0.168 (−0.227, 0.563)	−0.218 (−0.712, 0.276)
Cer (d18:0/24:1)	0.973 (0.826, 1.146)	−0.650 (−2.425, 1.125)	0.362 (−1.863, 2.588)
ChE (22:5n6)	1.017 (0.982, 1.053)	0.198 (−0.185, 0.582)	−0.113 (−0.595, 0.369)
ChE (22:5n3)	1.010 (0.984, 1.036)	0.096 (−0.184, 0.376)	−0.209 (−0.557, 0.140)
ChE (22:4)	1.115 (1.002, 1.240) *	0.537 (−0.642, 1.716)	−0.537 (−2.015, 0.940)
TAG (54:6)	0.999 (0.994, 1.005)	−0.051 (−0.112, 0.010)	−0.003 (−0.080, 0.075)

Adjusted for age, sex, BMI, smoking, LDL-C, SBP, and family history of hypertension. *, *p* < 0.05; **, *p* < 0.01.

## Data Availability

The data will be available on request.

## Supplementary Materials

The following supporting information can be downloaded at: https://www.mdpi.com/article/10.3390/nu17071289/s1, Figure S1. Mediation model. Figure S2. Metabolite levels in the plasma of the participants. Table S1. Internal standard of 11 metabolites. Table S2. Information on the detection limits and quantification limits of the six metabolites. Table S3. Associations of Metabolites with SSBP, ∆MAP_1_, and ∆MAP_2_ with and without FDR correction Table S4. Sensitivity analysis of metabolites and SSBP. Table S5. ROC analysis of metabolite in diagnosing SSBP.

## Abbreviations

The following abbreviations are used in this manuscript:

SSBP	Salt sensitivity of blood pressure
SS	Salt-sensitive
SR	salt-resistant
SSH	Salt-sensitive hypertension
BP	Blood pressure
MAP	Mean arterial pressure
ΔMAP_1_	Mean arterial pressure change during the acute saline load period
ΔMAP_2_	Mean arterial pressure change during the diuresis shrinkage period
BMI	Body mass index
ROC	Receiver operating characteristic
AUC	Area under the curve
eGFR	estimated glomerular filtration rate
