# Supplementary material for: Associations of Metabolites Related Salt Sensitivity of Blood Pressure and Essential Hypertension in Chinese Population: The EpiSS Study
Source: Nutrients. 2025 Apr 7;17(7):1289. doi: 10.3390/nu17071289 (PMC11990569; doi:10.3390/nu17071289)
Supplement: Supplementary file 1 [file nutrients-17-01289-s001.zip › Table S1.pdf]

Table S1. Internal standard of 11 metabolites.

Metabolites	Internal standards	CAS	Manufacturer / Country
N(6)-Methyllysine	L-Glutamine-d5	14341-78-7	Toronto, Canada
L-Glutamine	L-Glutamine-d5	14341-78-7	Toronto, Canada
L-Lactic acid	L-Lactic acid-d3	285979-84-2	Toronto, Canada
L-Malic acid	L-Lactic acid-d3	285979-84-2	Toronto, Canada
AcCa (20:3)	IS-AcCa (20:4)-d3	—	Merck，USA
PC (16:1/14:0)	IS-PC15:0/18:1d7	2097561-16-3	Merck，USA
Cer (d18:0/24:1)	IS-CER-18:1/24:1-d7	1840942-16-6	Merck，USA
ChE (22:5n6)	IS-ChE-18:1-d7	83199-47-7	Merck，USA
ChE (22:5n3)	IS-ChE-18:1-d7	83199-47-7	Merck，USA
ChE (22:4)	IS-ChE-18:1-d7	83199-47-7	Merck，USA
TAG (54:6)	IS-TAG-15:0/18:1d7	2097561-17-4	Merck，USA

Abbreviations: CAS, chemical abstracts service; AcCa, acylcarnitine; PC, phosphatidylcholines; Cer, ceramide; ChE, cholesteryl ester; TAG, triacylglycerol.
