# Supplementary material for: Associations of Metabolites Related Salt Sensitivity of Blood Pressure and Essential Hypertension in Chinese Population: The EpiSS Study
Source: Nutrients. 2025 Apr 7;17(7):1289. doi: 10.3390/nu17071289 (PMC11990569; doi:10.3390/nu17071289)
Supplement: Supplementary file 1 [file nutrients-17-01289-s001.zip › Table S2.pdf]

Table S2. Information on the detection limits and quantification limits of the six metabolites.

Metabolites	LOD(μg/L)	LOQ(μg/L)
N(6)-Methyllysine	50	200
L-Glutamine	500	1000
L-Lactic acid	5000	10000
L-Malic acid	50	50
13(S)-HODE	1	5
9(S)-HODE	1	2

Abbreviations: LOD, limit of detection; LOQ, limit of quantification.
