# Supplementary material for: Associations of Metabolites Related Salt Sensitivity of Blood Pressure and Essential Hypertension in Chinese Population: The EpiSS Study
Source: Nutrients. 2025 Apr 7;17(7):1289. doi: 10.3390/nu17071289 (PMC11990569; doi:10.3390/nu17071289)
Supplement: Supplementary file 1 [file nutrients-17-01289-s001.zip › Table S3.pdf]

Table S3. Associations of Metabolites with SSBP, ΔMAP_1, and ΔMAP_2 with and without FDR correction.

Metabolites	SSBP		ΔMAP_1		ΔMAP_2	
	p	*p*-FDR	*p*	*p*-FDR	*p*	*p*-FDR
N(6)-Methyllysine	0.236	0.614	0.148	0.589	0.592	0.841
L-Glutamine	0.001**	0.008**	0.178	0.589	0.246	0.640
L-Lactic acid	0.637	0.828	0.362	0.589	0.999	0.999
L-Malic acid	0.186	0.603	0.993	0.993	0.233	0.640
13(S)-HODE	0.519	0.749	0.544	0.589	0.061	0.640
9(S)-HODE	0.872	0.872	0.436	0.589	0.171	0.640
AcCa (20:3)	0.416	0.749	0.455	0.589	0.488	0.792
PC (16:1/14:0)	0.036*	0.213	0.406	0.589	0.388	0.792
Cer (d18:0/24:1)	0.743	0.872	0.475	0.589	0.750	0.887
ChE (22:5n6)	0.358	0.749	0.313	0.589	0.647	0.841
ChE (22:5n3)	0.462	0.749	0.503	0.589	0.244	0.640
ChE (22:4)	0.049*	0.213	0.374	0.589	0.478	0.792
TAG (54:6)	0.840	0.872	0.106	0.589	0.940	0.999

*, $p < 0.05$; **, $p < 0.01$.
