# Supplementary material for: Associations of Metabolites Related Salt Sensitivity of Blood Pressure and Essential Hypertension in Chinese Population: The EpiSS Study
Source: Nutrients. 2025 Apr 7;17(7):1289. doi: 10.3390/nu17071289 (PMC11990569; doi:10.3390/nu17071289)
Supplement: Supplementary file 1 [file nutrients-17-01289-s001.zip › Table S4.pdf]

Table S4. Sensitivity analysis of metabolites and SSBP.

Metabolites	Model 1		Model 2		Model 3		Model 4	
	OR (95% CI)	*p*	OR (95% CI)	*p*	OR (95% CI)	*p*	OR (95% CI)	*p*
N(6)-Methyllysine	1.020 (0.987, 1.054)	0.234	1.022 (0.990, 1.055)	0.187	1.022 (0.989, 1.055)	0.193	1.021 (0.989, 1.054)	0.208
L-Glutamine	0.998 (0.997, 0.999)	0.001	0.998 (0.997, 0.999)	<0.001	0.998 (0.997, 0.999)	<0.001	0.998 (0.996, 0.999)	<0.001
L-Lactic acid	1.035 (0.895, 1.197)	0.644	1.089 (0.941, 1.260)	0.257	1.088 (0.936, 1.266)	0.276	1.063 (0.921, 1.227)	0.406
L-Malic acid	1.015 (0.989, 1.041)	0.266	1.022 (0.996, 1.048)	0.097	1.022 (0.996, 1.048)	0.101	1.021 (0.995, 1.047)	0.118
13(S)-HODE	0.997 (0.985, 1.010)	0.658	0.999 (0.986, 1.011)	0.856	0.998 (0.985, 1.011)	0.795	0.997 (0.985, 1.010)	0.666
9(S)-HODE	1.000 (0.985, 1.015)	0.991	1.001 (0.986, 1.017)	0.868	1.001 (0.986, 1.017)	0.908	1.000 (0.985, 1.015)	0.990
AcCa (20:3)	0.916 (0.767, 1.095)	0.337	0.886 (0.744, 1.055)	0.177	0.885 (0.738, 1.061)	0.189	0.909 (0.764, 1.082)	0.285
PC (16:1/14:0)	1.039 (1.003, 1.078)	0.038	1.031 (0.994, 1.069)	0.109	1.033 (0.995, 1.073)	0.090	1.034 (0.998, 1.072)	0.069
Cer (d18:0/24:1)	0.995 (0.845, 1.171)	0.949	0.987 (0.841, 1.158)	0.876	0.983 (0.835, 1.157)	0.837	0.976 (0.831, 1.146)	0.769
ChE (22:5n6)	1.015 (0.979, 1.052)	0.418	1.013 (0.979, 1.049)	0.455	1.014 (0.979, 1.05)	0.437	1.015 (0.980, 1.051)	0.396
ChE (22:5n3)	1.009 (0.983, 1.035)	0.518	1.009 (0.984, 1.035)	0.494	1.009 (0.984, 1.035)	0.486	1.010 (0.984, 1.036)	0.466
ChE (22:4)	1.110 (0.996, 1.237)	0.061	1.091 (0.980, 1.214)	0.115	1.098 (0.984, 1.224)	0.099	1.101 (0.990, 1.225)	0.078
TAG (54:6)	0.999 (0.994, 1.005)	0.847	1.000 (0.994, 1.005)	0.956	1.000 (0.994, 1.005)	0.943	1.000 (0.994, 1.005)	0.903

Model 1 was adjusted for age, sex, BMI, smoking, LDL-C, and family history of hypertension.

Model 2 was adjusted for age, sex, BMI, smoking, LDL-C, DBP and family history of hypertension.

Model 3 was adjusted for age, sex, BMI, smoking, LDL-C, SBP, DBP, and family history of hypertension.

Model 4 was adjusted for age, sex, BMI, smoking, LDL-C, MAP, and family history of hypertension.
