# Supplementary material for: Associations of Metabolites Related Salt Sensitivity of Blood Pressure and Essential Hypertension in Chinese Population: The EpiSS Study
Source: Nutrients. 2025 Apr 7;17(7):1289. doi: 10.3390/nu17071289 (PMC11990569; doi:10.3390/nu17071289)
Supplement: Supplementary file 1 [file nutrients-17-01289-s001.zip › Table S5.pdf]

Table S5. ROC analysis of metabolite in diagnosing SSBP.

Metabolites	AUC	95% CI	*p*	Sensitivity	95% CI	Specificity	95% CI
**Single-metabolite model**							
L-Glutamine	0.766	(0.677, 0.855)	<0.001	0.759	(0.645, 0.873)	0.685	(0.561, 0.809)
PC (16:1/14:0)	0.715	(0.619, 0.812)	<0.001	0.463	(0.330, 0.596)	0.889	(0.805, 0.973)
ChE (22:4)	0.753	(0.661, 0.846)	<0.001	0.704	(0.582, 0.825)	0.741	(0.624, 0.858)
**Combined model**							
L-Glutamine	0.788	(0.703, 0.874)	<0.001	0.667	(0.541, 0.792)	0.870	(0.781, 0.960)
PC (16:1/14:0)							
ChE (22:4)							

Adjusted for age, gender, BMI, smoking, LDL-C, SBP and family history of hypertension.
