# Supplementary figures and images for: Associations of Metabolites Related Salt Sensitivity of Blood Pressure and Essential Hypertension in Chinese Population: The EpiSS Study
Source: Nutrients. 2025 Apr 7;17(7):1289. doi: 10.3390/nu17071289 (PMC11990569; doi:10.3390/nu17071289)

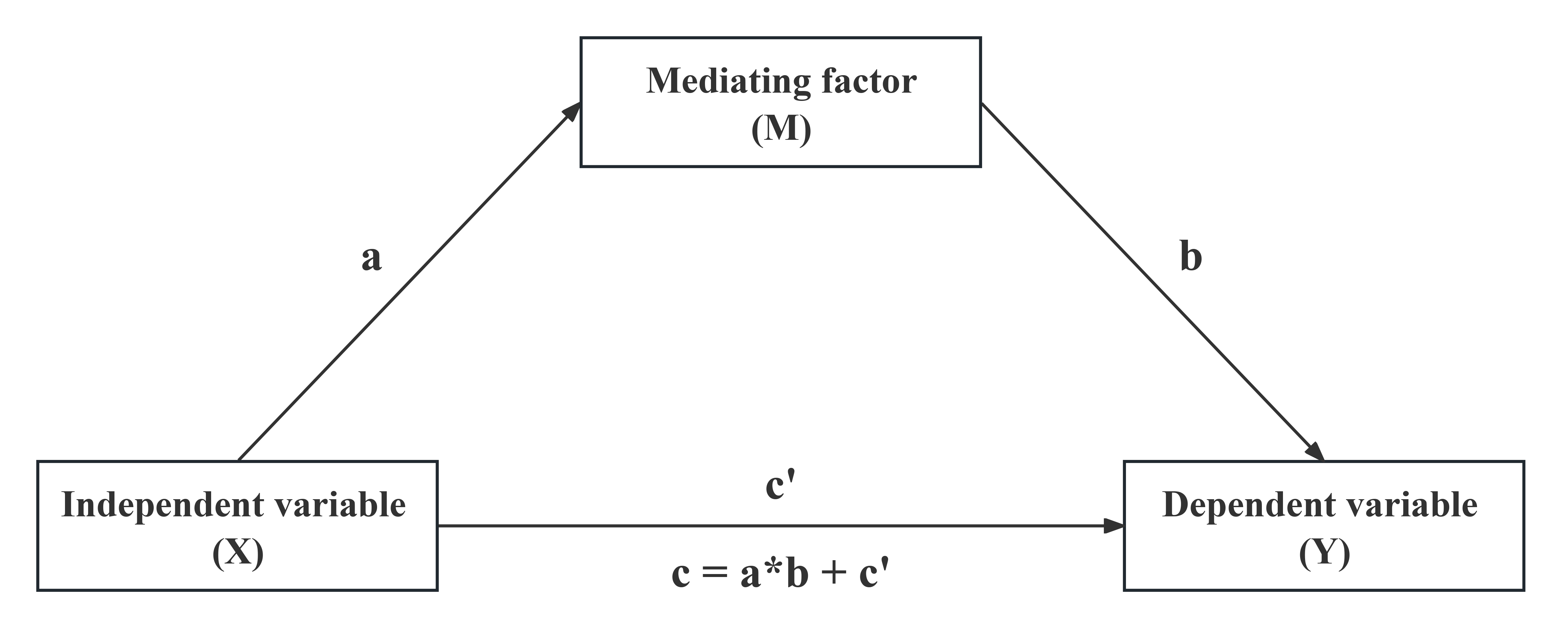

Supplement: Supplementary file 1 [file nutrients-17-01289-s001.zip › Figure S1.tif]

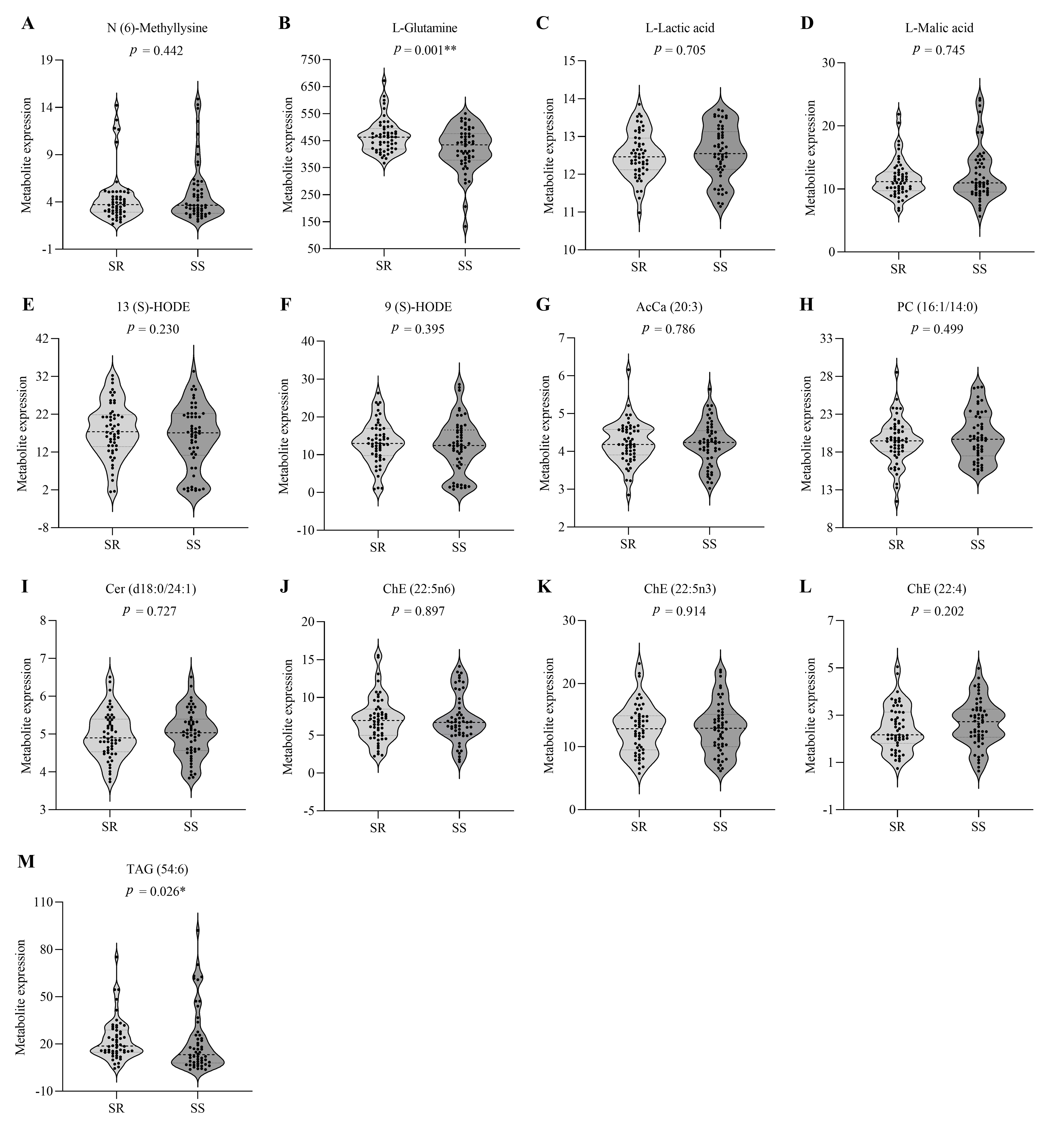

Supplement: Supplementary file 1 [file nutrients-17-01289-s001.zip › Figure S2.tif]
